# Prevalence and Factors Associated with Fixed-Dose Combination Antiretroviral Drugs Adherence among HIV-Positive Pregnant Women on Option B Treatment in Mpumalanga Province, South Africa

**DOI:** 10.3390/ijerph15010161

**Published:** 2018-01-20

**Authors:** Shandir Ramlagan, Karl Peltzer, Robert A. C. Ruiter, Nicole A. Barylski, Stephen M. Weiss, Sibusiso Sifunda

**Affiliations:** 1 HIV/Aids, STI and TB Unit, Human Sciences Research Council, Private Bag X41, Pretoria 0001, South Africa; kpeltzer@hsrc.ac.za (K.P.); ssifunda@hsrc.ac.za (S.S.); 2 Department of Work & Social Psychology, Maastricht University, P.O. Box 616, 6200 MD Maastricht, The Netherlands; r.ruiter@maastrichtuniversity.nl; 3 Department of Research and Innovation, University of Limpopo, Sovenga 0727, South Africa; 4 Miller School of Medicine, University of Miami, 1400 NW 10th Ave, Miami, FL 33136, USA; barylskin14@miami.edu (N.A.B.); sweiss2@med.miami.edu (S.M.W.)

**Keywords:** HIV/AIDS, pregnant, ARVs, adherence, Option B

## Abstract

The possibility for all babies to be born and remain HIV-negative for the first year of life is achievable in South Africa. HIV-positive mothers’ adherence to their antiretroviral medication is one of the crucial factors to achieve this target. Cross-sectional data were collected at 12 community health centres, over 12 months (2014–2015), from 673 HIV-positive women, less than 6 months pregnant, attending antenatal care, and on Option B treatment. Adherence measures included the Adults AIDS Clinical Trials Group (AACTG) four-day measure, as well as the Visual Analog Scale (VAS) seven-day measure. Bivariate analyses and multivariate logistic regressions are presented. 78.8% of respondents were adherent on AACTG, while 68.8% reported VAS adherence. Bivariate analyses for increased adherence show significant associations with older age, less/no alcohol usage, disclosure of HIV status, higher HIV knowledge, no desire to avoid ARV side effects, low stigma, and low depression. AACTG showed a negative association with intimate partner violence. Multivariable logistic regression on AACTG and VAS adherence rates resulted in unique contributions to increased adherence of older age, less/no alcohol usage, higher HIV knowledge, lack of depression, and non-disclosure. Programs targeting closer side effect monitoring, HIV disclosure, pre-natal depression, alcohol intake, and HIV knowledge need consideration.

## 1. Introduction

Globally an estimated 36.7 million people were living with HIV in the year 2016; 1.8 million became newly infected and 1 million lost their lives to AIDS-related diseases [1]. Sub-Saharan Africa remains the global epicentre of this epidemic where nearly 71% of the global HIV infections occurred in 2014 with an estimated 24.7 million people [2]. The latest South African population-based HIV prevalence and incidence study estimated HIV prevalence at 12.2% or 6.4 million people, and HIV incidence at 1.1% or 469,000 people [3]. Because of the high incidence and prevalence of HIV in South Africa, antiretroviral (ARV) adherence of HIV-infected pregnant women on fixed dose combinations (FDC) represents an important prevention strategy.

South Africa has approximately 3.7 million HIV-positive people on ARV treatment [4], which is more than any other country in the world [1]. Each year, over 1.4 million HIV infected women give birth, with 91% residing in sub-Saharan Africa [5], leading to a high potential rate of mother-to-child transmission (MTCT) of HIV. Currently, MTCT rates in South Africa are 1.5% at 6 weeks and 4.3% at 18 months [4]. In order to reduce MTCT rates, pregnant women need to adhere to their ARVs, as women who are adherent are less likely to transmit their HIV to their baby [6,7]. There were two ARV treatment plans in place in South Africa during 2014–2015 and include Option A and Option B. This was due to the phasing out of Option A for the better Option B. Options B is known as the test and treat model as the protocol states that as soon as an individual tests HIV-positive, they can be placed on ARV treatment immediately while they wait for their CD4 count [8,9]. This is important because, in Option A, ARV treatment could only begin once a CD4 count result is obtained. In a resource poor setting like Mpumalanga Province, South Africa, this could take many months. In the Option B treatment protocol, ARVs will continue to be provided to the mother after the cessation of breastfeeding only if the mothers’ health requires it. 

An important setback, as noted in Malawi, was that those who initiated their ARV regimen during pregnancy were twice as likely to miss their first follow-up visit [10]. These missed appointments and follow-ups decrease the effectiveness of the treatment [10,11]. Another factor to impact ARV adherence negatively is alcohol abuse [7,12]. It would seem that as alcohol intake increases, people become less adherent. Furthermore, a large number of pregnant women do not want to deal with both the side effects of the ARVs and the normal side effects of pregnancy at once, so many interrupt treatment or postpone initiation of it to avoid side effects [13] such as exacerbated nausea, vomiting, and gastro-intestinal issues, to state a few [14]. In early pregnancy, 70–85% of women experience morning sickness, and in later pregnancy many experience heartburn [14]. To avoid exacerbation of these symptoms by ARV medications, adherence during this period can be reduced [14,15]. Finally, the lack or presence of social support greatly affects the way a woman, especially a pregnant women, follows her HIV treatment plan [14]. In South Africa especially, the social stigma of being infected with HIV is a common reason of sub-optimal adherence [15,16,17]. On the other hand, disclosure of HIV status and treatment support by a partner has been shown to be associated with good adherence [18]. Those who receive proper social support tend to have reduced depression scores and better ARV adherence [17,19]. 

However, adherence can also be increased as a result of the pregnancy, as maternal desire to protect the unborn child is one of the greatest motivators of good adherence [18]. Factors associated with better adherence include strong beliefs about the necessity of medication [20,21] as well as older age where the older the person is, the more likely it is that the person will adhere to the ARVs [12,14]. When women understood the importance of the viral load and CD4 test results and received basic prevention of mother-to-child transmission (PMTCT) services, their emotional outlooks improved and they were more apt to take actions to protect their unborn child [19]. 

There is extremely limited data on ARV adherence during pregnancy [14]. In terms of the Option B regime and pregnant women, there are no data available. The purpose of this study was to examine the prevalence and factors associated with fixed-dose combination antiretroviral drugs among HIV-infected pregnant women on Option B treatment in South Africa. 

## 2. Materials and Methods 

### 2.1. Sample and Procedure 

Cross-sectional data of 673 HIV-positive pregnant women were collected over a 12-month period from April 2014 to March 2015. This baseline data is part of the Protect Your Family randomised controlled trial [22] conducted in 12 community health centres (CHCs) in Gert Sibande and Nkangala districts in Mpumalanga province, South Africa. The participants were all less than six months pregnant at the time of the baseline interview, and were attending antenatal care (ANC) at the study CHCs. During the baseline period, all HIV-positive pregnant women in Mpumalanga province attending public ANC were offered Option B treatment. As per the South African PMTCT protocol [23] and the South African antiretroviral treatment guidelines [24], pregnant women recruited during our study period, were provided tenofovir (TDF) + emtricitabine (FTC) or lamivudine (3TC) + efavirenz (EFV) as fixed dose combinations. 

Potential participants were referred to trained study fieldworkers by ANC nurses at the study CHCs utilising the indicators of less than six months pregnant and HIV-positive. Fieldworkers screened the potential participant adding the following indicators: (1) being 18 years and older, (2) having a current partner (not necessarily the father of the baby), and (3) having not been recruited in this study at another CHC. If the participant met all screening indicators, they were invited to participate and, if interested, the participants were then introduced to the study information sheet and consent form in English, isiZulu, or seSotho. Once the consent process was completed, participants completed the baseline assessment utilising the Audio Computer-Assisted Self-Interview (ACASI) technology [25], which was used to increase confidentiality and self-disclosure and ensure the inclusion of women across all literacy levels. 

The ACASI software was loaded onto touch screen Lenovo ThinkPad X230 tablet/laptop (with 180° swivel screen). Logitech h150 stereo over-ear headphones were utilised to maintain privacy. All participants were given brief training on the usage of the laptop touchscreen, headphones, and ACASI software. The fieldworkers assisted the participants to complete the initial demographic variables to ensure they were comfortable with ACASI, and all other questions were then completed individually by the participant with the fieldworker in the room to assist should a question or problem arise and to collect the completed questionnaire once the participant had completed it. Participants were compensated with R50.00 (South African Rand = US$4.72) for time and transportation.

### 2.2. Measures

The ACASI questionnaire requested information on sociodemographic characteristics, HIV related issues, intimate partner violence, stigma, and depression. How these factors might impact adherence to ARVs was the main outcome measure.

#### 2.2.1. Socio-Demographic Characteristics

Questions regarding socio-demographic characteristics included age at last birthday; language—Nguni (isiZulu, isiXhosa, isiSwati, and isiNdebele) and others (Afrikaans, English, Sesotho, Sepedi, Setswana, Tshivenda, Xitsonga, other European, and other); educational attainment (completed Grade 11 or less and completed Grade 12 or more); partner status (are couples living together or not); employment status (not employed versus employed, volunteer or student); income per month in South African Rand (ZAR) where the South African Government child grant was R310.00 (UD$29.27) during the study period; number of children; if this pregnancy was planned or unplanned; and alcohol use of more than 2 drinks on at least one occasion in the previous four weeks (yes vs. no).

#### 2.2.2. HIV-Related Characteristics

Questions regarding HIV-related characteristics asked participants for the following: whether HIV was diagnosed during their current pregnancy (yes vs. no); the period of time spent on ARV medication (<12 months vs. ≥12 months); whether the their HIV status was disclosed to anyone (yes vs. no); whether any of their children were HIV-positive (yes vs. no); whether their partner was HIV-positive (yes vs. no); and, in terms of male involvement, whether their male partner knew what happened in the antenatal clinic (yes vs. no). If the respondent had no children, they were not asked if any of their children were HIV-positive, and the question of partners’ status was not asked if the respondent stated “no” or “don’t know” to the question, “Has your partner or spouse been tested for HIV?” 

HIV knowledge was also attained, and questions included HIV transmission, condom use, PMTCT, and AIDS, and was assessed using 18 items adapted from an AIDS-related knowledge scale [26]. HIV knowledge was scored for the number of correct responses (Yes or No), with Don’t Know responses scored as incorrect (score 1 or 0). The possible range of scores 0 to 18 was expressed as the percentage correct, meaning that the higher the score, the more HIV knowledge a respondent had. The scores were dichotomized using a cutoff of greater than or less than and equal to 13/18 correct [27]. Cronbach’s Alpha reliability for the scale was α = 0.74.

#### 2.2.3. Intimate Partner Violence (IPV)

*A*n adapted 18-item version of that Conflict Tactics Scale 18 (CTS-18) [28,29] was used to assess psychological and physical aggression, as well as reasoning. Respondents were presented with 18 conflict situations such as “discussed the issue calmly,” “cried,” “did something to spite you,” “threw something at you,” “slapped you,” etc. and were asked to rate the number of times she and her partner may had engaged in such conflict in the previous month on a seven point scale of 0 (never) to 6 (more than 20 times). A higher score is indicative of increased IPV. Cronbach’s Alpha reliability for the scale was α = 0.84.

#### 2.2.4. Aids-Related Stigma

The nine-item AIDS-Related Stigma Scale (ARSS) [30] was used to assess externalised stigma using items such as “People who have AIDS are dirty,” “People who have AIDS should be ashamed,” etc. This is a dichotomous scale using a score of 0 (disagree) to 1 (agree). Due to internal reliability of the original nine-item scale, question four, a reverse scored item, was removed to increase reliability. Scores on the scale range from 0 to 8, where higher scores indicate greater levels of stigma. Cronbach’s Alpha reliability for the scale was α = 0.73. 

#### 2.2.5. Depression

The 10-item Edinburgh Postnatal Depression Scale 10 (EPDS-10) [31] asked participants to rate how often they had experienced different symptoms associated with depression in the previous seven days. Questions included “I have been able to laugh and see the funny side of things,” “I have felt sad or miserable,” etc. Scores ranged from 0 to 30, where the higher the score, the more the likelihood of depression being experienced. This paper utilised the validated cut-off score for South African populations at score 12 [32]. Cronbach’s Alpha reliability for the scale was α = 0.66. 

#### 2.2.6. Adherence

Two self-report instruments were used to assess adherence, namely the Adult AIDS Clinical Trials Group (AACTG) [20], and an adapted version of the ARV adherence Visual Analog Scale (VAS) [33]. The AACTG adherence measure is a four day recall instrument asking “How many doses did you miss: (1) yesterday, (2) day before yesterday, (3) three days ago, and (4) four days ago.” Given that the current ARV regiment in South Africa is one combination pill per day, a dose would translate to one ARV pill per day. Participants were recorded as adherent if they had taken all (100%) of their ARV medication over the last four days. In order to understand non-adherence, the AACTG instrument provides 14 possible reasons a person may have for missing their medication and includes but is not limited to the following: were away from home, had too many pills to take, felt sick or ill, wanted to avoid side effects, and so on [20]. Responses were dichotomized to yes vs. no. 

The second instrument utilised in this study was an adapted version of the VAS which included ARV usage over the previous seven days and asked participants if they had taken “None,” “Half,” or “All” of their medication in the previous seven days [33]. If participants reported that they had taken “All” of their medication in the previous seven days, they were recorded as “adherent” and coded as 1 in the statistical program. Participants were recorded as 0 if they too reported that they had taken “None” or “Half” of their medication on any day in the previous seven days.

### 2.3. Data Analysis

Software SPSS, version 24.0 (Statistical Product and Service Solutions, IBM, New York, NY, USA) was used for data analyses. Frequencies, means, and cross-tabulations were calculated to describe the sample. Reliability tests were done on the ARSS, EPDS, and IPV scales. A *t*-test was conducted on adherence measures and self-rated adherence measure. Bivariate analyses and multivariable logistic regressions were used to investigate associations between the outcomes ARV adherence and socioeconomic, HIV-related, and behavioural variables. Associations were considered significant at *p* < 0.05. All statistically significant variables in the bivariate analyses were included in the multivariable model. Multi-collinearity was tested. 

### 2.4. Ethical Approval

Ethics approval for the study was granted by the HSRC Research Ethics Committee with Protocol No REC 4/21/08/13 as well as the University of Miami Miller School of Medicine Institutional Review Board (IRB ID: 20130238 (CR00006122)). Approval was also obtained from the Mpumalanga Provincial Government: Department of Health. 

## 3. Results

### Sample Characteristics

A total of 673 HIV-positive pregnant women, with a mean age of 28 years old (SD = 5.73), were entered into this study. The majority (58%) were between the ages of 18 and 29 years old. In terms of sample characteristics, Table 1 shows the majority (81%) of respondents spoke an Nguni language, which includes Xhosa, Zulu, Swati and Ndebele, while only 29% had completed school. All participants in this study had a partner, as this was a study inclusion requirement, yet the majority (62%) of respondents did not live with their partners. Unemployment was high, as 78% of respondents reported that they were unemployed with 33% of respondents receiving less than R310 monthly income. Only 21% or 139 respondents stated that they do not have other children with just over half (53%) reported that this current pregnancy was unplanned. In terms of alcohol intake, 14% of our pregnant respondents reported drinking two or more alcoholic drinks on at least one occasion in the previous four weeks. 

Table 1 also shows ARV adherence on the two adherence measures across sociodemographic variables. Overall, 21% of respondents reported to be non-adherent over the four-day recall AACTG scale, while 31% reported to be non-adherent on the seven-day recall VAS. With all the sociodemographic variables, the VAS always reported 10% higher non-adherence of respondents when compared to the AACTG scale. In terms of those couples “not living together,” 23% and 34% reported non-adherence with AACTG and VAS, respectively, and 19% and 27% of those “living together” reported non-adherence with AACTG and VAS, respectively. Twenty-five percent and 36% of respondents who receive a monthly income of less than R310 reported non-adherence with AACTG and VAS, respectively; 19% and 29% of those with a higher monthly income reported non-adherence with AACTG and VAS, respectively. Of those that stated that this was an unplanned pregnancy, 23% and 32% reported non-adherence with AACTG and VAS, respectively, while 20% and 30% of those who reported that this was a planned pregnancy reported non-adherence with AACTG and VAS, respectively. In this survey of HIV-positive pregnant women, 14% of respondents reported drinking two or more alcoholic drinks on at least one occasion in the previous four weeks. Of these, 39% on AACTG and 49% on VAS reported non-adherence. 

Table 2 shows that just over half (54%) of all respondents reported that they were diagnosed with HIV during this pregnancy, and three quarters had initiated ARV medication less than 12 months prior. A quarter of respondents stated that they had an HIV-positive partner, and 58% stated that their partner was involved in the pregnancy and are aware of what occurs during clinic visits (male involvement). In terms of disclosing their HIV status to anyone, 72% of respondents stated that they had disclosed their HIV status. Just over three-fifths demonstrated high knowledge of HIV and PMTCT issues. Of those respondents that have children (79%), a low percentage (4.2%) reported that they had an HIV-positive child. In terms of IPV, stigma, and depression, a high 20% of respondents reported experiencing IPV, 41% reporting stigma, and 49% reporting depression.

In terms of adherence, Table 2 shows that 23% and 33% of those respondents who had been on ARV medication for less than 12 months reported non-adherence with AACTG and VAS, respectively, while those 25% who had been on ARV medication for a year or longer reported 15% and 29% non-adherence with AACTG and VAS, respectively. Those who had disclosed their HIV status to anyone, 18% and 28% reported non-adherence with AACTG and VAS, respectively, while 29% and 40% of those who had not disclosed it reported non-adherence with AACTG and VAS, respectively. Twenty-seven percent and 39% of respondents that demonstrated no to low HIV knowledge reported non-adherence with AACTG and VAS, respectively, while 18% and 27% of those that demonstrated high HIV knowledge reported non-adherence with AACTG and VAS, respectively. In terms of IPV, of those respondents who stated that they experienced IPV, 28.8% and 37.9% reported non-adherence with AACTG and VAS, respectively, yet of those who reported not experiencing IPV, 19% and 30% reported non-adherence with AACTG and VAS, respectively. Of those who experienced internalised stigma, 25% and 36% reported non-adherence with AACTG and VAS, respectively, while 16% and 28% of respondents who reported no internalised stigma reported non-adherence with AACTG and VAS, respectively. In terms of respondents who reported higher depression scores, 27% and 37% reported non-adherence with AACTG and VAS, respectively, yet 16% and 26% of those with lower depression scores reported non-adherence with AACTG and VAS, respectively.

In both the AACTG and VAS scales, the *t*-test with self-rated adherence found people that are adherent perceive their adherence to be better than people that are not adherent. When investigating the reasons for non-adherence, the logistic regression for the AACTG and VAS adherence indicator and the 14 possible reasons for missing medication [20] shows only 12% variance meaning that the reasons are not predictors of adherence but merely excuses for not taking ARVs. The reason “Desire to avoid side effects,” which was answered Yes by 13.5% (*N* = 91) of respondents, showed significance (*p* =< 0.05) for both AACTG and VAS, pointing out that people may avoid their ARVs due to side effects. 

In Table 3, the bivariate logistic regression, the both the AACTG and the VAS adherence measure shows associations between age, alcohol usage of less than two alcoholic drinks on at least on one occasion in the previous four weeks, disclosure of HIV status to anyone, higher HIV knowledge, fear of side effects of the ARV medication, low levels of stigma, and depression. Additionally, the bivariate logistic regression with AACTG showed association with more than 12 months on ARVs as well as no IPV. All variables that were found to be statistically significant in the bivariate analysis were put into a multivariate model looking at associations for AACTG and VAS adherence (see Table 3). In the multivariate model, older age was associated with increased ARV adherence on both the AACTG and VAS measure, as well as drinking less than two alcoholic drinks on at least on one occasion in the previous four weeks. The multivariate model on both the AACTG and VAS measure goes on to show significant associations with disclosure of HIV status to anyone, higher HIV knowledge as well as no or low depression. Chances of non-adherence are shown with both the AACTG and VAS measure to increase due to a desire to avoid side effects of the ARV medication.

## 4. Discussion

The high non-adherence (21% AACTG non-adherence and 31% VAS non-adherence) to ARV medication by HIV-positive pregnant women is of serious concern. There were a few factors that were found to contribute to non-adherence. The intake of alcohol has been shown to decrease ARV adherence [7,12,14]; in this study, for those respondents that consumed less than two alcoholic drinks on at least on one occasion in the previous four weeks, adherence to ARVs was more likely than those that consumed more than two alcoholic drinks on one occasion. Alcohol is widely accepted as a disinhibitor, and this study shows that drinking less alcohol increases adherence to ARVs among pregnant women. It would seem that, as the pregnant women consume alcohol, they either forget about their ARVs, cannot take it due to the lack of privacy of their drinking location, or cannot take it due to the alcohol/ARV interaction. 

Non-adherence to ARVs was also more likely for those who wanted to avoid the side effects of the ARV medication. As mentioned earlier, early stages of pregnancy are characterized by morning sickness and late stages with heartburn [14], and these conditions could be exacerbated by the side effects of ARVs, which include but are not limited to nausea, vomiting, and gastro-intestinal issues [14]. To ease discomfort, it is easy to assume that some pregnant women may not adhere to the ARV treatment regime. 

Disclosure, or rather the lack of disclosure, of ones HIV-positive status to anyone was strongly associated with non-adherence of ARV medication. For those that do disclose, Futteman et al. (2010) and Nchenga et al. (2012) show that social support leads to decreased depression, which in turns leads to increased adherence to ARVs. Depression is a contributor to ARV adherence [7,14]; in rural Mpumalanga, we found that those who reported lower depression had better adherence to ARVs. The PMTCT protocol does make provisions for emotional well-being, where it is seen as protective to the mother and ultimately the unborn child [19]. It stands to reason that a pregnant woman with little or no depressive symptoms will be more likely to be adherent to her ARVs than a depressed pregnant woman. It must also be noted that the majority (75%) of the study respondents initiated Efavirenz-based ARV medication within the previous 12 months and that 54% were diagnosed HIV-positive during this pregnancy. These factors could contribute to depression. Interestingly, all of the above-mentioned contributing factors to ARV adherence could be related to the amount of HIV knowledge a respondent may or may not have. The study data clearly shows that non-adherence was related to no/low HIV knowledge. With knowledge, people are empowered to act to protect themselves and their unborn child [19].

An important factor for increased adherence to ARV was that of older age. The results indicate that respondents who were of an older age were more likely to be adherent than younger respondents in our sample [12,14] and seem to suggest increased adherence responsibility with age. 

## 5. Conclusions

In order to achieve a PMTCT goal of first reducing and then eliminating HIV transmission from mother to child, thereby ensuring all babies born are HIV-negative and remain that year for their first year of their lives, adherence to ARV is a necessary step. It is of great concern to find a high percentage of the pregnant HIV-positive respondents in this study to be non-adherent to their ARV medication. Older respondents were found to be more likely to be adherent then younger respondents. Participants who reported increased alcohol intake, those who showed no to lower HIV knowledge, those who wanted to avoid the ARV side effects, those who did not disclosure their HIV status, and those who showed increased depression were more likely to be non-adherent. 

Due to the findings, it is suggested that programs that encourage disclosure of one’s HIV and pregnancy status are still needed and need to be strengthened. It is also advised that programs targeting prenatal alcohol intake as well as programs designed to help mothers with prenatal depression as well as HIV depression also need to be investigated and encouraged. It is also advised that more, closer monitoring is needed with regard to the ARV side effects to mitigate its effects and increase adherence. More ongoing education campaigns are definitely needed and will assist with disclosure, alcohol intake, depression, ARV side effects, and any other barriers to better adherence to ARV treatment. More research is needed on adherence to ARV among different populations in South Africa and the world, as there is currently limited research.

## Figures and Tables

**Table 1:** Sample characteristics.

Variable	Sample	AACTG Adherence		VAS Adherence	
		Adherent	Non-Adherent (Missed at Least 1 Dose)	Adherent	Non-Adherent (Missed at Least 1 Dose)
**Socio-demographics**	*N* (%)	*N* (%)	*N* (%)	*N* (%)	*N* (%)
**All**	673 (100)	530 (78.8)	143 (21.2)	463 (68.8)	210 (31.2)
**Language**					
Nguni languages	546 (81.1)	432 (79.1)	114 (20.9)	373 (68.3)	173 (31.7)
Other languages	127 (18.9)	98 (77.2)	29 (22.8)	90 (70.9)	37 (29.1)
**Educational attainment**					
Grade 11 and less	481 (71.5)	386 (80.2)	95 (19.8)	340 (70.7)	141 (29.3)
Grade 12 or more	192 (28.5)	144 (75.0)	48 (25.0)	123 (64.1)	69 (35.9)
**Relationship status (couples)**					
Not living together	419 (62.3)	324 (77.3)	95 (22.7)	277 (66.1)	142 (33.9)
Living together	254 (37.7)	206 (81.1)	48 (18.9)	186 (73.2)	68 (26.8)
**Employment status**					
Not employed	527 (78.3)	416 (78.9)	111 (21.1)	363 (68.9)	164 (31.1)
Employed, Volunteer or Student	146 (21.7)	114 (78.1)	32 (21.9)	100 (68.5)	46 (31.5)
**Income (ZAR) per month**					
<R310	221 (32.8)	165 (74.7)	56 (25.3)	141 (63.8)	80 (36.2)
R311 or more	452 (67.2)	365 (80.8)	87 (19.2)	322 (71.2)	130(28.8)
**Number of children**					
None	139 (20.7)	109 (78.4)	30 (21.6)	95 (68.3)	44 (31.7)
One or more	534 (79.3)	421 (78.8)	113 (21.2)	368 (68.9)	166 (31.1)
**Unplanned pregnancy**					
Yes	356 (52.9)	276 (77.5)	80 (22.5)	242 (68.0)	114 (32.0)
No	317 (47.1)	254 (80.1)	63 (19.9)	221 (69.7)	96 (30.3)
**Alcohol use of more than 2 drinks on at least one occasion in the previous four weeks**					
No	581 (86.3)	474 (81.6)	107 (18.4)	416 (71.6)	165 (28.4)
Yes	92 (13.7)	56 (60.9)	36 (39.1)	47 (51.1)	45 (48.9)

**Table 2:** Health and behaviour characteristics.

Variable	Sample	AACTG Adherence		VAS Adherence	
		Adherent	Non-Adherent (Missed at Least 1 Day)	Adherent	Non-Adherent (Missed at Least 1 Day)
	*N* (%)	*N* (%)	*N* (%)	*N* (%)	*N* (%)
**All**	673 (100)	530 (78.8)	143 (21.2)	463 (68.8)	210 (31.2)
**Diagnosed with HIV in this pregnancy**					
No	308 (45.8)	247 (80.2)	61 (19.8)	214 (69.5)	94 (30.5)
Yes	365 (54.2)	283 (77.5)	82 (22.5)	249 (68.2)	116 (31.8)
**Time on ARV medication**					
<12 months	506 (75.2)	388 (76.7)	118 (23.3)	341 (67.4)	165 (32.6)
≥12 months	167 (24.8)	142 (85.0)	25 (15.0)	122 (73.1)	45 (26.9)
**Disclosure of HIV serostatus to anyone**					
No	188 (27.9)	133 (70.7)	55 (29.3)	113 (60.1)	75 (39.9)
Yes	485 (72.1)	397 (81.9)	88 (18.1)	350 (72.2)	135 (27.8)
**HIV-positive children**					
No or do not know	506 (94.8)	398 (78.7)	108 (21.3)	351 (69.4)	155 (30.6)
Yes	28 (5.2)	23 (82.1)	5 (17.9)	17 (60.7)	11 (39.3)
**HIV-positive partner**					
No or do not know	506 (75.2)	387 (76.5)	119 (23.5)	335 (66.2)	171(33.8)
Yes	167 (24.8)	143 (85.6)	24 (14.4)	128 (76.6)	39(23.4)
**Male involvement**					
No	281 (41.8)	217 (77.2)	64 (22.8)	188 (66.9)	93 (33.1)
Yes	392 (58.2)	313 (79.8)	79 (20.2)	275 (70.2)	117 (29.8)
**HIV knowledge**					
No/Low	257 (38.2)	187 (72.8)	70 (27.2)	158 (61.5)	99 (38.5)
High	416 (61.8)	343 (82.5)	73 (17.5)	305 (73.3)	111 (26.7)
**Intimate partner violence**					
No mild or No severe physical violence	541 (80.4)	436 (80.6)	105 (19.4)	381 (70.4)	160 (29.6)
Mild or severe physical violence	132 (19.6)	94 (71.2)	38 (28.8)	82 (62.1)	50 (37.9)
**AIDS-Related Stigma**					
No	400 (59.4)	326 (81.5)	74 (18.5)	288 (72.0)	112 (28.0)
Yes	273 (40.6)	204 (74.7)	69 (25.3)	175 (64.1)	98 (35.9)
**Depression**					
No/Low	345 (51.3)	290 (84.1)	55 (15.9)	256 (74.2)	89 (25.8)
Yes/High	328 (48.7)	240 (73.2)	88 (26.8)	207 (63.1)	121 (36.9)

**Table 3:** Association of demographic, socioeconomic, health, and behaviour characteristics to ARV non-adherence.

Variable	AACTG Non-Adherence		VAS Non-Adherence	
	Cr OR (95% CI) * *p*	Adj OR (95% CI) * *p*	Cr OR (95% CI) * *p*	Adj OR (95% CI) * *p*
**Age (scale)**	1.06 (1.02–1.10) * *p =* 0.001	1.05 (1.02–1.09) * *p* = 0.006	1.04 (1.01–1.08) * *p* = 0.004	1.04 (1.01–1.08) * *p* = 0.009
**Language**				
Nguni languages	1.12 (0.71–1.78)		0.89 (0.58–1.35)	
Other languages				
**Educational attainment**				
Grade 12 or more	0.74 (0.50–1.10)		0.74 (0.52–1.05)	
Grade 11 and less				
**Relationship status**				
Not living together	0.80 (0.54–1.172)		1.40 (0.99–1.98)	
Living together				
**Employment status**				
Not employed	1.05 (0.67–1.64)		1.02 (0.69–1.15)	
Employed, student, volunteer				
**Income (ZAR) per month**				
<R310	0.70 (0.48–1.03)		1.41 (1.00–1.98)	
R311 or more				
**Number of children**				
None	0.98 (0.62–1.54)		0.97 (0.652–1.46)	
One or more				
**Unplanned pregnancy**				
No	1.17 (0.81–1.69)		1.08 (0.78–1.50)	
Yes				
**Alcohol use of 2 or more drinks on at least one occasion in the previous four weeks**				
No	0.35 (0.22–0.56) * *p* = 0.000	0.43 (0.26–0.71) * *p* = 0.001	0.41 (0.27–0.65) * *p* = 0.000	0.49 (0.31–0.79) * *p* = 0.003
Yes				
**Diagnosed with HIV in this pregnancy**				
No	1.17 (0.81–1.70)		1.06 (0.76–1.47)	
Yes				
**Time on ARV medication**				
<12 months	1.73 (1.08–2.77) * *p* = 0.023	1.29 (0.77–2.18) *p* = 0.340	1.31 (0.89–1.94)	
≥12 months				
**Desire to avoid side effects**				
No	0.36 (0.23–0.57) * *p* = 0.000	0.42 (0.25–0.79) * *p* = 0.001	0.36 (0.23–0.57) * *p* = 0.000	0.39 (0.24–0.63) * *p* = 0.000
Yes				
**Disclosure of HIV serostatus to anyone**				
No	1.87 (1.26–2.76) * *p* = 0.002	1.76 (1.16–2.68) * *p* = 0.008	1.72 (1.21–2.45) * *p* = 0.003	1.72 (1.18–2.50) * *p* = 0.004
Yes				
**HIV-positive children**				
No	0.80 (0.30–2.16)		1.47 (0.67–3.20)	
Yes				
**HIV-positive partner**				
No or Don’t Know	0.68 (0.35–1.32)		0.74 (0.42–1.31)	
Yes				
**Male involvement**				
No	1.17 (0.81–1.70)		1.16 (0.84–1.62)	
Yes				
**HIV knowledge**				
No/Low	1.76 (1.21–2.55) * *p* = 0.003	1.46 (0.98–2.17) *p* = 0.060	1.72 (1.24–2.40) * *p* = 0.001	1.47 (1.04–2.09) * *p* = 0.030
High				
**Intimate partner violence**				
No	0.60 (0.39–0.92) * *p* = 0.019	0.79 (0.49–1.27) *p* = 0.337	1.45 (0.98–2.16)	
Yes				
**Stigma (Scale)**	0.67 (0.46–0.97) * *p* = 0.035	0.79 (0.56–1.25) *p* = 0.389	0.69 (0.50–0.97) * *p* = 0.030	0.82 (0.58–1.16) *p* = 0.254
**Depression**				
No/Low	0.52 (0.35–0.76) * *p* = 0.001	0.63 (0.42–0.948) * *p* = 0.023	0.60 (0.43–0.83) * *p* = 0.002	0.71 (0.50–1.00) * *p* = 0.049
Yes/High				

* 95% significance.
